# Study on Stability and Fluidity of HPMC-Modified Gangue Slurry with Industrial Validation

**DOI:** 10.3390/ma18153461

**Published:** 2025-07-23

**Authors:** Junyu Jin, Xufeng Jin, Yu Wang, Fang Qiao

**Affiliations:** 1 College of Coal Engineering, Shanxi Datong University, Datong 037009, China; 230857002143@sxdtdx.edu.cn (X.J.); 240857002121@sxdtdx.edu.cn (Y.W.); 240857002113@sxdtdx.edu.cn (F.Q.); 2 The Cultivation Base of Shanxi Key Laboratory of Coal Mine Water Jet Technology and Equipment, Shanxi Datong University, Datong 037009, China

**Keywords:** gangue slurry, response surface methodology (RSM), hydroxypropyl methylcellulose (HPMC), stability, fluidity, industrial validation

## Abstract

HPMC, regulating slurry properties, is widely used in cement-based materials. Research on the application of HPMC in gangue slurry is still in its early stages. Moreover, the interactive effects of various factors on gangue slurry performance have not been thoroughly investigated. The work examined the effects of slurry concentration (X_1_), maximum gangue particle size (X_2_), and HPMC dosage (X_3_) on slurry performance using response surface methodology (RSM). The microstructure of the slurry was characterized via scanning electron microscopy (SEM) and polarized light microscopy (PLM), while low-field nuclear magnetic resonance (LF-NMR) was employed to analyze water distribution. Additionally, industrial field tests were conducted. The results are presented below. (1) X_1_ and X_3_ exhibited a negative correlation with layering degree and slump flow, while X_2_ showed a positive correlation. Slurry concentration had the greatest impact on slurry performance, followed by maximum particle size and HPMC dosage. HPMC significantly improved slurry stability, imposing the minimum negative influence on fluidity. Interaction terms X_1_X_2_ and X_1_X_3_ significantly affected layering degree and slump flow, while X_2_X_3_ significantly affected layering degree instead of slump flow. (2) Derived from the RSM, the statistical models for layering degree and slump flow define the optimal slurry mix proportions. The gangue gradation index ranged from 0.40 to 0.428, with different gradations requiring specific slurry concentration and HPMC dosages. (3) HPMC promoted the formation of a 3D floc network structure of fine particles through adsorption-bridging effects. The spatial supporting effect of the floc network inhibited the sedimentation of coarse particles, which enhanced the stability of the slurry. Meanwhile, HPMC only converted a small amount of free water into floc water, which had a minimal impact on fluidity. HPMC addition achieved the synergistic optimization of slurry stability and fluidity. (4) Field industrial trials confirmed that HPMC-optimized gangue slurry demonstrated significant improvements in both stability and flowability. The optimized slurry achieved blockage-free pipeline transportation, with a maximum spreading radius exceeding 60 m in the goaf and a maximum single-borehole backfilling volume of 2200 m^3^.

## 1. Introduction

Coal gangue is a solid waste generated during coal mining and dressing by washing, accounting for approximately 10–20% of coal production [1]. Based on data from the International Energy Agency (IEA) and BigMint, global coal production in 2024 reached 9.068 billion tons (with China, India, and Indonesia contributing 52.5, 11.5, and 9.2%, respectively), corresponding to an estimated coal gangue production of 1.2–1.8 billion tons. Current comprehensive utilization methods for coal gangue include power generation (practiced in the United States and China), alumina extraction (Germany and Japan), building material manufacturing (China and the United States), as well as land reclamation and underground backfilling (China and Indonesia) [2,3,4,5,6,7]. Developed countries have achieved a comprehensive utilization rate of coal gangue exceeding 90%. However, developing nations such as China, India, and Indonesia exhibit relatively lower utilization rates, still relying primarily on stockpiling [8,9].

As the world’s largest coal producer, China has accumulated 7 billion tons of coal gangue, with an annual increase of 700 million tons [10,11,12,13]. The stockpiling of coal gangue occupies vast amounts of land and pollutes the atmospheric environment [14,15,16]. Efficient treatment of coal gangue has become an inevitable requirement for promoting the construction of a low-carbon and environmentally friendly society in China under the current dual carbon strategy [17,18,19].

Gangue slurry backfilling, as an effective method for coal gangue disposal, operates on the following technical principles [20,21,22,23,24]. Coal gangue is mixed with water at a predetermined ratio after crushing to a specific particle size to prepare a non-cemented high-concentration slurry with a solids content of approximately 75%. This slurry is then transported via pipelines to the goaf for backfilling. Since this technology allows backfilling to proceed without interfering with coal mining processes and requires no cementitious materials, it achieves high backfilling efficiency and low operational costs [25,26,27,28]. However, this technology faces a critical challenge—the difficulty in simultaneously optimizing slurry stability and fluidity in practical engineering applications. Specifically, improved slurry stability increases flow resistance, which negatively impacts pipeline transport distance. Enhanced slurry fluidity reduces stability, raising the risk of sedimentation and pipeline blockage [29,30].

HPMC, as a polymeric additive demonstrating notable advantages in modifying slurry performance, has been widely used in cement-based materials such as mortar and concrete. Wang et al. [31] discovered that HPMC addition strengthens the flocculated network structure within the paste slurry, which enhances its stability with only a slight effect on fluidity. Zhao et al. [32] observed that an appropriate amount of HPMC maintains high fluidity while improving viscosity and segregation resistance. Yang et al. [33] concluded that HPMC enhances the homogeneity and integrity of cemented coal-mine backfill slurry with only a minor impact on pipeline flow resistance. HPMC increases slurry viscosity but has little influence on its yield stress. (Yin et al. [34]; Ebru et al. [35]; Parham et al. [36]) demonstrated that the incorporation of HPMC exerts a positive impact on 3D printing materials, primarily manifested in the enhancement of slurry extrudability, shape stability, and buildability.

Based on the application of HPMC in cement-based materials, some researchers have explored its effects on the performance of coal gangue slurry. Wang et al. [37] investigated the influence of compound additives on coal gangue slurry properties. Approximately 0.5% water reducer and 0.06% HPMC can reduce bleeding while maintaining fine fluidity. Ma et al. [38] discovered that a mixture of 0.06% HPMC and 0.08% air-entraining agent can achieve the optimal balance between slurry stability and fluidity.

In summary, adding an appropriate amount of HPMC to coal gangue slurry significantly improves stability while having only a minor negative impact on fluidity. These research findings have advanced the application of gangue slurry backfill technology. However, existing studies exhibit notable limitations: (1) They primarily focus on single-factor influences on gangue slurry performance, neglecting interactive effects between factors. (2) There is a lack of systematic analysis regarding HPMC’s action mechanism in gangue slurry systems.

RSM enables comprehensive consideration of systematic errors and multi-factor interactions through systematic experimental design, multivariate quadratic equation fitting, and model analysis. Response values are predicted, which provides a holistic evaluation of slurry performance evolution [39]. In light of this, the work employed RSM to investigate the influence of slurry concentration, maximum gangue particle size, and HPMC dosage on the stability and fluidity of gangue slurry. Response surface regression models for layering degree and slump flow concerning these factors were established, with an optimal slurry mix ratio proposed. The microstructure of the slurry was characterized by SEM and PLM, while LF-NMR quantified its water distribution, collectively elucidating the mechanism by which HPMC enhances gangue slurry properties. Industrial trials were conducted to validate the regulatory effect of HPMC on gangue slurry performance, providing both theoretical foundations and practical support for engineering applications. This work systematically evaluates HPMC’s role in gangue slurry for the first time, combining RSM optimization with microstructural analysis to bridge lab-to-field scalability gaps.

## 2. Materials and Methods

### 2.1. Materials

**Figure 1:**
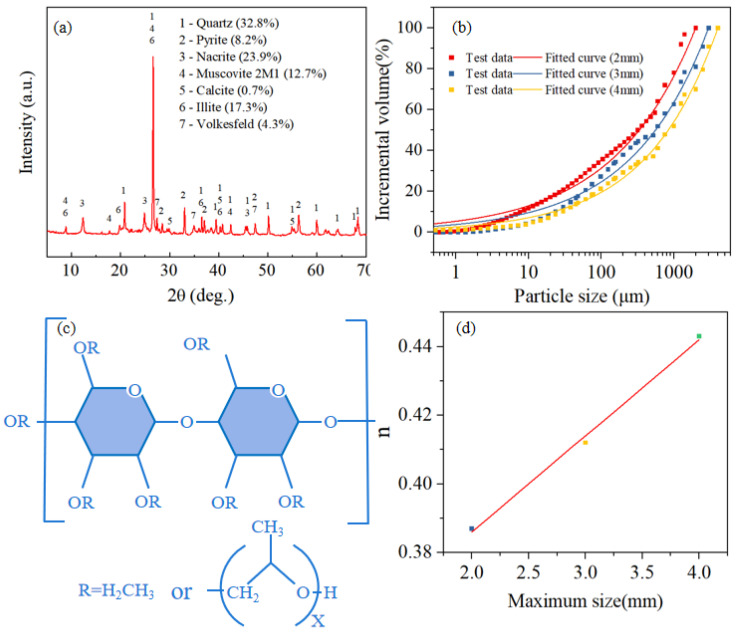
Characteristics of raw materials (**a**) XRD-CG, (**b**) PSD-CG, (**c**) MS-HPMC, (**d**) Fitting curves of maximum particle size and n.

Raw materials used in the experiments included coal gangue (CG), additives, and water. Coal gangue was sourced from Wangjiata Mine in Ordos City, Inner Mongolia, China. XRD analysis reveals that its primary mineral composition consisted of quartz, paragonite, and illite, with these three minerals accounting for ≥70% of the total content (Figure 1a). Gangue was crushed using a hammer crusher into three maximum particle sizes of 2, 3, and 4 mm. The particle size distribution (PSD) curves (Figure 1b) are accurately fitted (R^2^ > 0.98) using the Talbol gradation formula (Equation (1)), with corresponding gradation index n of 0.443, 0.412, and 0.387, respectively. Taking the maximum particle size of gangue as the horizontal axis and n as the vertical axis, a graph is plotted (Figure 1d). The relationship between the two is established through fitting (Equation (2)). The additive selected is HPMC, whose non-ionic aqueous solution exhibits thickening, suspending, and stabilizing effects [25]. Figure 1c presents its molecular structure (MS) formula. The experimental water used is municipal tap water compliant with the GB 5749-2022 standard [40], with pH of 7.2 ± 0.3, which meets the experimental requirements.

(1) $$y=100{\left(\frac{d_{i}}{d_{\max}}\right)}^{n}$$

where $d_{i}$ represents different particle sizes; $d_{max}$ represents the maximum particle size in the material; y represents the percentage of particles smaller than size $d_{i}$.

(2) $$y=0.33+0.028\times x\;(R^{2}=0.996)$$

### 2.2. Experimental Design and Sample Preparation

Slurry performance is influenced by multiple factors, including main effects and interaction effects. Traditional single-factor analysis neglects interactions between factors, which makes it difficult to systematically reveal the variation of slurry performance. In contrast, RSM can simultaneously analyze multi-factor interactions and enable continuous prediction and optimization of response values [39].

The work employed the Box-Behnken Design (BBD) to construct a three-factor, three-level experiment, investigating the effects of X_1_ (76–78%), X_2_ (2–4 mm), and X_3_ (0–0.06%) on layering degree (Y_1_) and slump flow (Y_2_). Table 1 lists the experimental factor levels and coding scheme.

During sample preparation, HPMC was first dissolved in water at 1% concentration and left to stand for 24 h to ensure complete dissolution. According to the experimental design scheme, coal gangue and water were sequentially added into a mixer and stirred until homogeneous. Subsequently, the HPMC solution was incorporated and mixed again to ensure uniformity. A portion of the prepared slurry was taken for slurry stability and fluidity tests, while the remaining slurry was poured into standard Φ50 × 100 mm molds. The samples were cured at room temperature for 7 days before demolding, followed by continued curing for 28 days for microstructural analysis of the hardened filling material.

### 2.3. Experimental Methods

#### 2.3.1. Slurry Stability Test

Slurry stability was evaluated using the layering degree test, which combined a layering degree cylinder and a consistency meter. The layering degree cylinder consisted of an upper section (200 × Φ200 mm) and a lower section (100 × Φ200 mm). The consistency meter included a test cone (H145 × Φ75 mm), a graduated scale, and a container cylinder (H180 × Φ150 mm). Freshly mixed slurry was poured into the layering degree cylinder and allowed to stand for 2 h. Following the JGJ/T 70-2009 standard [41], slurry consistency in the upper and lower sections was measured separately. The difference in consistency between the two sections was recorded as the layering degree.

#### 2.3.2. Slurry Fluidity Test

Slurry fluidity was characterized by slump flow. The test apparatus included a mini slump cone with an upper diameter of 50 mm, a lower diameter of 100 mm, and a height of 150 mm. The test procedure followed the GB/T 2419-2005 standard [42]. Each slurry mixture was tested three times, and the average was taken to minimize experimental error.

#### 2.3.3. Determining Water Distribution in Slurry

Hydrogen protons in water molecules possess nuclear spin characteristics. Low-Field Nuclear Magnetic Resonance (LF-NMR) applies radiofrequency pulses to induce energy level transitions in hydrogen protons. Relaxation time T_2_, characterizing the distribution of water molecules in the material, was recorded as the high-energy-state protons return to the low-energy state after pulse termination. The relaxation time corresponding to the peak value in the T_2_ curve reflects the water freedom degree—longer relaxation time indicates a higher water freedom degree. The peak area quantitatively represents the relative volume content of water in different states. The tests were conducted using a MesoMR23-060H-I NMR analyzer (Suzhou Niumag Corporation, Suzhou, China) with the magnetic field strength of 0.3 ± 0.03 T, an operating frequency of 12 MHz, and 60 mm probe coils. Data were obtained based on the Carr-Purcell-Meiboom-Gill (CPMG) sequence.

#### 2.3.4. Microscopic Morphology Observation of Materials

The microscopic morphology of naturally hardened slurry was observed using a Scanning Electron Microscope (SEM, Model JSM-6700F). Hardened slurry samples were polished into a 1 cm^3^ cube, and then observed under vacuum conditions after coating with conductive film. A Murzider 9224 Polarized Light Microscopy (PLM) (Mashidi (Dongguan) Technology Co., Ltd., Dongguan, China) was employed to examine the microscopic morphology of fine particles in the diluted slurry state and complement SEM observations. Dilute the slurry with water at a ratio of 1:200. Allow the mixture to stand for 2 min. Extract the upper suspension and place a droplet on a glass slide. Position the slide on the PLM stage and adjust the magnification for observation.

## 3. Results and Discussion

### 3.1. Experimental Results and Response Surface Regression Model

Table 2 lists the measured Y_1_ and Y_2_ of slurries with different ratios. A multivariate quadratic nonlinear model (Equation (3)) is employed to fit the 15 sets of experimental data from Table 2. Equations (4) and (5) show the established response surface model between the response values of Y_1_ and Y_2_ and the influencing factors of X_1_, X_2_, and X_3_, respectively. The response surface regression analysis demonstrates high model accuracy, with R^2^ of 0.9981 and 9978 for the layering degree model and slump flow models, respectively.

(3) $$Y=\beta_{0}+\sum_{i=1}^{k}\beta_{i}X_{i}+\sum_{i=1}^{k}\beta_{ii}X_{i}^{2}+\sum_{i=1}^{k}\beta_{ij}X_{i}X_{j}+\varepsilon \;(i=1,2,\cdots ,n-1)$$

(4) $$\begin{array}{c} Y_{1}=-913.0+30.9X_{1}-20.3X_{2}+620.1X_{3}+0.35X_{1}X_{2}-10.0X_{1}X_{3}\\ +7.5X_{2}X_{3}-0.25X_{1}^{2}-0.42X_{2}^{2}-189.8X_{3}^{2} \end{array}$$

(5) $$\begin{array}{c} Y_{2}=-132476+3525.2X_{1}-964.0X_{2}+3419.4X_{3}+13.5X_{1}X_{2}-50.0X_{1}X_{3}\\ +100.0X_{2}X_{3}-23.42X_{1}^{2}-7.92X_{2}^{2}-6574.1X_{3}^{2} \end{array}$$

### 3.2. Variance Analysis of the Response Surface Regression Model

A significance test is conducted on the model through variance analysis (Table 3) to verify the reliability of the response surface regression model. F-values of the layering degree and slump flow models are 3537.4 and 1331.97, respectively, both significantly greater than F_0.05_(9, 5) ≈ 4.77, with *p*-value for both models were far below 0.0001, which indicates that the models are highly reliable.

**Figure 2:**
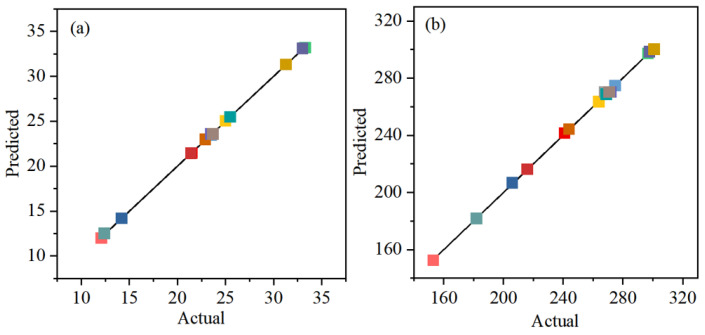
Predicted and actual values (**a**) Layering degree, (**b**) Slump flow.

Scatter plots are generated to compare the predicted values (*y*-axis) and actual values (*x*-axis) of the laying degree and slump flow (Figure 2). Scatter points are evenly distributed near the Y = X line, exhibiting an approximately linear relationship. High consistency between the model’s predicted values and the actual values demonstrates that both models possess high reliability for predicting slurry performance.

### 3.3. Influence of Single Factors on Slurry Performance

In Table 3, the *p*-values of X_1_, X_2_, and X_3_ are all less than 0.0001, demonstrating significant effects of these factors on both slurry layering degree and slump flow. The relationship F(X_1_) > F(X_2_) > F(X_3_) indicates that the primary-to-secondary order of influence on the layering degree and slump flow is: slurry concentration > maximum gangue particle size > HPMC dosage.

**Figure 3:**
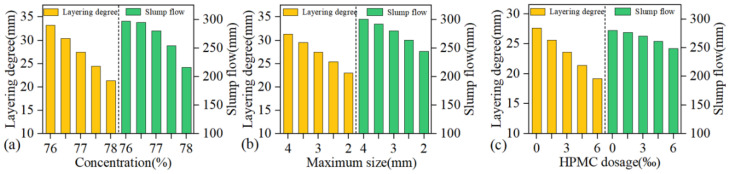
Influence of single factors on slurry performance (**a**) concentration, (**b**) maximum size, (**c**) HPMC dosage.

Figure 3a (X_2_ = 3 mm and X_3_ = 0%), Figure 3b (X_1_ = 77% and X_3_ = 0%), and Figure 3c (X_1_ = 77% and X_2_ = 3 mm) illustrate the effects of slurry concentration (X_1_), maximum gangue particle size (X_2_), and HPMC dosage (X_3_) on slurry performance. Increased slurry concentration, reduced maximum gangue particle size, or increased HPMC dosage enhance slurry stability but reduce its fluidity. Fluidity loss rate L is defined as the ratio of the slump flow range to the layering degree range under the same factor (L = Δslump flow/Δlayering degree), providing a quantitative assessment of the combined impact of each factor on slurry stability and fluidity. Based on data in Figure 3, L(X_1_) = 6.84, L(X_2_) = 6.74, and L(X_3_) = 3.76. Improved slurry stability by increasing slurry concentration, reducing maximum gangue particle size, or adding HPMC dosage negatively affects fluidity in the following order: slurry concentration > maximum gangue particle size > HPMC dosage. Notably, incorporating small HPMC dosage significantly enhances slurry stability and maintains acceptable fluidity, demonstrating a superior comprehensive regulatory advantage.

### 3.4. Multi-Factor Interaction Effects

#### 3.4.1. Slurry’s Layering Degree

**Figure 4:**
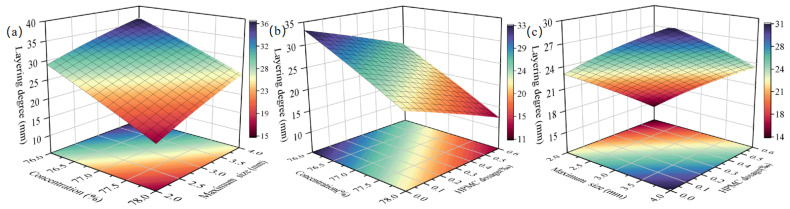
Effect of interaction terms on layering degree (**a**) X_1_X_2_ (X_3_ = 0%) (**b**) X_1_X_3_ (X_2_ = 3 mm) (**c**) X_2_X_3_ (X_1_ = 77%).

In Table 3, the *p*-values of X_1_X_2_, X_1_X_3_, and X_2_X_3_ in layering degree models are less than 0.05, indicating a highly significant effect on layering degree. Figure 4a–c show the relationships between the interaction terms—X_1_X_2_ (X_3_ = 0%), X_1_X_3_ (X_2_ = 3 mm), and X_2_X_3_ (X_1_ = 77%)—and layering degree, respectively.

In Figure 4a, with X_3_ = 0%, as X_1_ increases from 76 to 78%, the layering degree decreases from 29.0 to 16.5 mm (a reduction of 12.5 mm) at X_2_ = 2 mm and from 36.6 to 25.5 mm (a reduction of 11.1 mm) at X_2_ = 4 mm. The influence of X_1_ on the layering degree becomes stronger as X_2_ decreases. In Figure 4b, with X_2_ = 3 mm, as X_1_ increases from 76 to 78%, the layering degree decreases by 11.8 mm at X_3_ = 0% and by 10 mm at X_3_ = 0.06%. The lower the X_3_, the more sensitive the layering degree is to changes in X_1_. In Figure 4c, with X_1_ = 77%, as X_3_ increases from 0 to 0.06%, the layering degree decreases by 7.9 mm at X_2_ = 4 mm and by 8.8 mm at X_2_ = 2 mm. The smaller the X_2_, the greater the impact of X_3_ on the layering degree.

#### 3.4.2. Slurry Fluidity

**Figure 5:**
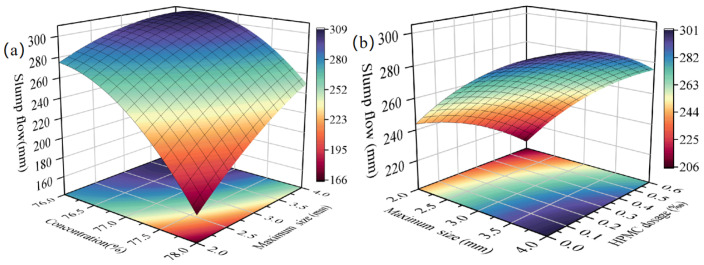
Effect of interaction terms on fluidity (**a**) X_1_X_2_ (X_3_ = 0%) (**b**) X_2_X_3_ (X_1_ = 77%).

Table 3 shows that X_1_X_2_ and X_2_X_3_ (*p* < 0.05) significantly affect the slurry’s slump flow, while X_1_X_3_ (*p* > 0.05) does not. Figure 5a (X_3_ = 0%) and Figure 5b (X_1_ = 77%) illustrate the influences of X_1_X_2_ and X_2_X_3_ on layering degree, respectively.

In Figure 5a, with X_3_ = 0%, as X_1_ increases from 76 to 78%, slump flow decreases by 54 mm at X_2_ = 4 mm and by 108 mm at X_2_ = 2 mm. The smaller the X_2_, the more sensitive the slump flow is to changes in X_1_. In Figure 5b, with X_1_ = 77%, as X_2_ decreases from 4 to 2 mm, slump flow decreases by 56 mm at X_3_ = 0% and by 68 mm at X3 = 0.06%. Higher X_3_ enhances the influence of X_2_ on slump flow.

### 3.5. Mix Proportion Validation of Gangue Slurry

**Figure 6:**
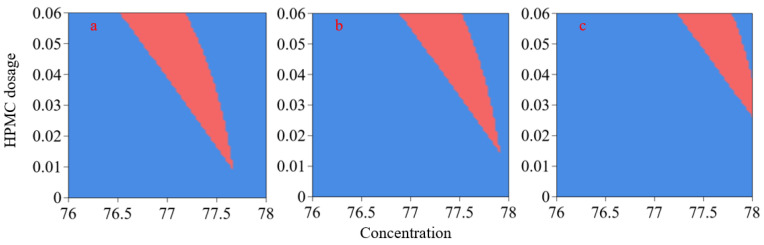
Reasonable concentration and HPMC dosages under different maximum particle sizes: (**a**) 2.5 mm; (**b**) 3.0 mm; (**c**) 3.5 mm.

Fine fluidity and stability are a prerequisite for ensuring normal backfilling of gangue slurry. Slurry segregation can be avoided when the layering degree is ≤ 20 mm. When slump flow > 220 mm, the slurry meets the required flowability. Based on these constraints, the optimal mix proportion of gangue slurry was determined using the optimization module in Design-Expert 13. The results demonstrate that when the maximum gangue particle size exceeds 3.5 mm, the feasible slurry range becomes narrow, which makes it impractical for actual slurry preparation. Additionally, current equipment exhibits low efficiency and excessive wear when gangue is crushed to a maximum particle size of 2 mm. Therefore, the maximum particle size should be controlled within 2.5–3.5 mm. Based on Equation (2), the corresponding gradation index (n) is 0.40–0.428. Figure 6 illustrates reasonable slurry concentration and HPMC dosage for the maximum particle sizes of 2.5, 3, and 3.5 mm. The red regions represent feasible solutions that satisfy the layering degree (≤20 mm) and slump flow (>220 mm).

Four ratios within the red region are selected for laboratory testing (Table 4) to verify the accuracy of model predictions. The prediction errors for both layering degree and slump flow of the four slurries are less than 5%, confirming the reliability of the response surface model.

### 3.6. Microscopic Morphology Analysis of Slurry

#### 3.6.1. SEM Observation

**Figure 7:**
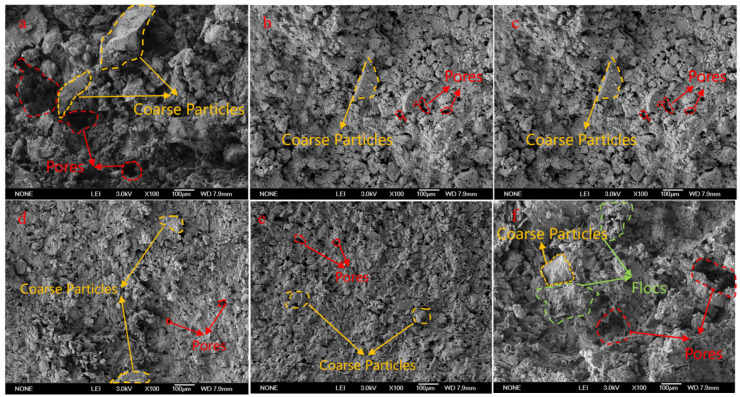
Microscopic morphology of slurries under SEM: (**a**) X_1_ = 77%, X_2_ = 4 mm, (**b**) X_1_ = 77%, X_2_ = 3 mm, (**c**) X_1_ = 77%, X_2_ = 2 mm, (**d**) X_1_ = 78%, X_2_ = 3 mm, (**e**) X_1_ = 79%, X_2_ = 3 mm, (**f**) X_1_ = 77%, X_2_ = 3 mm, and ((**a**–**e**) X_3_ = 0%, (**f**) X_3_ = 0.05%).

SEM is used to investigate the effects of slurry concentration, maximum gangue particle size, and HPMC dosage on the microstructure of consolidated slurry (Figure 7). As slurry concentration increases, slurry density increases (Figure 7b,d,e). This is attributed to the increased solid content, promoting a tighter packing of gangue particles, which compresses pore sizes. The reduction in the maximum gangue particle size (Figure 7a–c) induces a transformation in the slurry structure. Specifically, the structure evolves from a loose configuration characterized by large pores and dominated by coarse particles to a denser arrangement featuring smaller pores and composed of finer particles. This transition is due to the decrease in the maximum particle size, which increases the number of particles and shortens the average distance between particles.

HPMC addition significantly alters the slurry structure (Figure 7b,f). Fine particles are dispersed independently with clear contours without HPMC. However, fine particles adhere to each other after HPMC addition, which forms a flocculent network structure permeating the slurry system. The multi-point active groups on the HPMC polymer chains can simultaneously bind to unsaturated bonds on the surfaces of different gangue particles, acting as an adsorption bridge. This promotes the connection between particles and the formation of a network structure [43].

#### 3.6.2. PLM Observations

**Figure 8:**
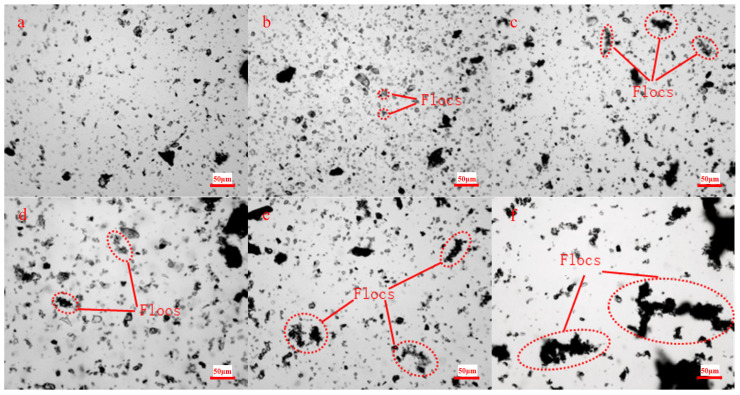
Microscopic morphology of slurries under the orthogonal PLM: (**a**) X_1_ = 77%, X_2_ = 4 mm, (**b**) X_1_ = 77%, X_2_ = 3 mm, (**c**) X_1_ = 77%, X_2_ = 2 mm, (**d**) X_1_ = 78%, X_2_ = 3 mm, (**e**) X_1_ = 79%, X_2_ = 3 mm, (**f**) X_1_ = 77%, X_2_ = 3 mm, and((**a**–**e**) X_3_ = 0%, (**f**) X_3_ = 0.05%).

A PLM is used to observe the slurry diluted 200 times (Figure 8), which can be used to intuitively obtain the floc (network) structure in the slurry. Only a small number of small flocs exist in the slurry without HPMC. Moreover, the size and quantity of these flocs slightly increase with the decreased maximum gangue particle size or increased slurry concentration. Reduced maximum gangue particle size increases the fine particle content with high surface activity, while increased slurry concentration shortens the interparticle distance. Both factors promote the interaction between fine particles, which increases the number and size of flocs. However, the limited self-flocculation ability of gangue particles underdevelops the floc structure.

Multi-scale large flocs are formed in the slurry after HPMC addition, which highly corresponds to the floc network structure observed by SEM. HPMC molecules significantly enhance the interparticle interaction through the adsorption-bridging effect.

### 3.7. Water Distribution by LF-NMR

**Figure 9:**
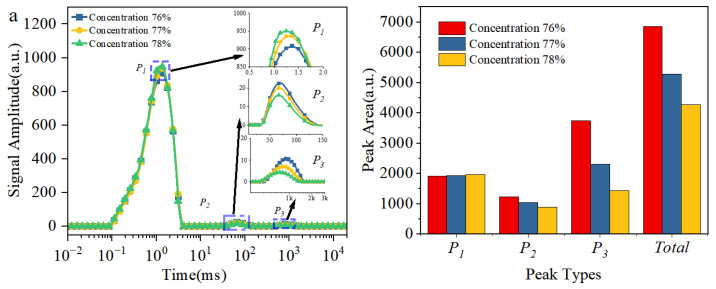

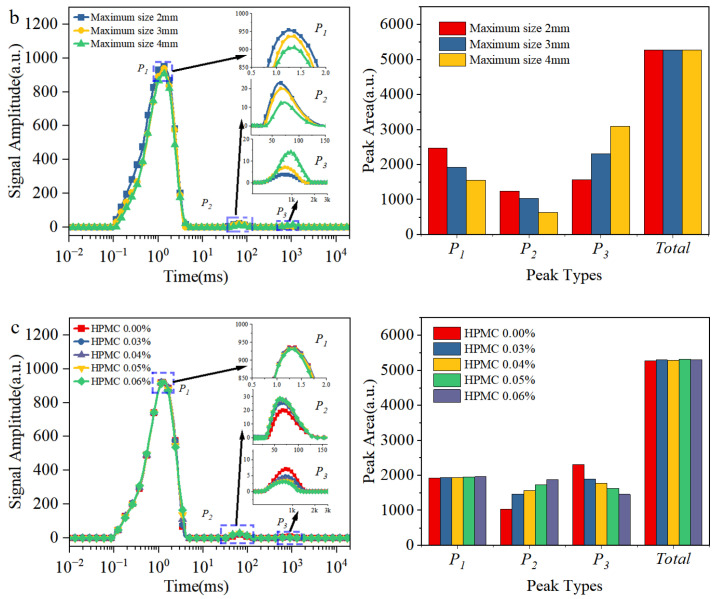
Influences of different factors on the water distribution (**a**) X_1_ (X_2_ = 3 mm and X_3_ = 0%), (**b**) X_2_ (X_1_ = 77% and X_3_ = 0%), (**c**) X_3_ (X_1_ = 77% and X_2_ = 3 mm).

Figure 9 shows the T_2_ relaxation spectra of slurries with different ratios and the calculated areas of each relaxation peak. The T_2_ spectra of all slurry groups exhibit three distinct peaks, which are sequentially labeled from left to right as P_1_, P_2_, and P_3_. These peaks correspond to water molecules in different states of occurrence. P_1_ (0.1–5 ms) represents adsorbed water, referring to water molecules tightly adsorbed on particle surfaces due to the electrostatic effects of the electrical double layer. Adsorbed water exhibits quasi-solid characteristics macroscopically under strong surface confinement, with almost no flowability. P_2_ (10–200 ms) denotes floc water, referring to moisture trapped inside floc structures. Its mobility lies between that of free water and adsorbed water. P_3_ (500–2000 ms) corresponds to free water, which is distributed in the gaps between particles or flocs. This interconnected water contributes to optimal flowability [43].

For the slurry without HPMC, as slurry concentration increases from 76 to 78%, the relaxation peaks of P_1_, P_2_, and P_3_ in the T_2_ spectrum all shift to the left (Figure 9a left). The pore structure of the slurry becomes more compact with increasing concentration, which significantly restricts water mobility. Quantitative analysis of the relaxation peaks (Figure 9a right) reveals a sharp decrease in free water by 2298 a.u., indicating substantial loss due to higher concentration. Floc water decreased by 335 a.u., reflecting that reduced interparticle spacing leads to compression of the intra-floc void space. Adsorbed water shows a slight increase of 57 a.u., suggesting that double-layer adsorbed water is less affected by concentration changes. The increase in concentration primarily reduces the free water content by compressing pore space, while its impact on surface-adsorbed water and floc water is relatively limited. When the maximum gangue particle size is reduced from 4 to 2 mm, the three relaxation peaks in the T_2_ spectrum shift leftward (Figure 9b left). However, the moisture migration characteristics differ significantly from those influenced by concentration control. Free water decreases by 1520 a.u., with 60.1% (914 a.u.) transforming into adsorbed water and 39.9% (606 a.u.) converting into floc water, in Figure 9b right. The reduction in maximum particle size increases the fine particle content, which expands the total solid surface area and enhances the particle surface’s binding effect on water molecules. Simultaneously, the higher fine particle content promotes floc development, which strengthens the encapsulation of free water and causes partial conversion into floc water. Particle size regulation achieves moisture redistribution through a synergistic surface binding–floc encapsulation mechanism.

When the HPMC content increases from 0 to 0.6%, the range and area of P_1_ in the T_2_ relaxation spectrum remain largely unchanged, in Figure 9c. HPMC has minimal influence on adsorbed water, which exists in a stable state within the slurry. The formation of adsorbed water primarily relies on the electrical double-layer effect on the surface of gangue particles. This effect mainly depends on the physicochemical properties of the particle surface and the inherent volume of the liquid phase. The ranges of the P_2_ and P_3_ peaks show little variation. However, the P_2_ peak area increases by 840 a.u., while the P_3_ peak area decreases by 851 a.u., HPMC does not significantly change the mobility of floc water or free water; however, it facilitates the conversion of part of the free water into floc water. HPMC addition enhances interparticle interactions, which promotes the development of floc structures within the slurry. More free water is transformed into floc water after encapsulation.

### 3.8. HPMC Action Mechanism

**Figure 10:**
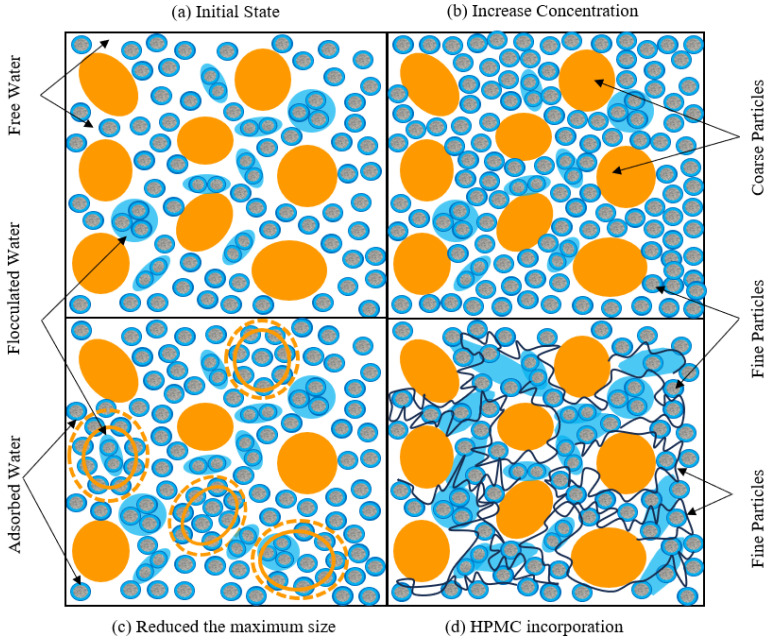
Internal structure of gangue slurry (**a**) Initial state, (**b**) Increased concentration, (**c**) Reduced maximum gangue particle size, (**d**) HPMC incorporation.

Gangue slurry, as a mixed system of multi-scale particles and water (Figure 10a), initially exhibits a typical unstable structure. Gangue particles are randomly distributed, with their surfaces enveloped by an adsorbed water film stabilized by the electrical double layer. Some fine particles form flocs through mutual interactions, with floc water trapped inside these structures. A significant amount of free water occupies the gaps between flocs and particles. Coarse particles are prone to sedimentation under gravity, which results in poor slurry stability.

Traditional approaches enhance slurry stability by increasing slurry concentration or reducing the maximum particle size, yet both exhibit significant drawbacks. Their common mechanism relies on decreasing interparticle spacing to amplify the frictional force between gangue particles, which improves stability. However, this process drastically reduces the free water content, severely compromising slurry fluidity, in Figure 10b,c.

The slurry system undergoes remarkable structural refinement after HPMC addition (Figure 10d). HPMC connects fine particles through an adsorption-bridging effect to form a 3D floc network. This network acts as a mechanical framework supporting coarse particles, which inhibits sedimentation and improves slurry stability. Crucially, HPMC converts only a minimal portion of free water into floc water, preserving overall fluidity. Slurry stability and fluidity are synergistically optimized.

The effectiveness of HPMC is constrained by the inherent properties of the slurry, and its efficacy depends on appropriate slurry concentration and reasonable particle gradation. (1) The slurry should maintain a critical concentration to ensure the continuity of the flocculated network structure. (2) The fine particle content ought to meet the minimum carrier requirement for HPMC’s bridging effect. If slurry concentration is insufficient or the proportion of fine particles is too low, blindly increasing HPMC dosage will yield limited improvement and significantly raise backfilling costs. Therefore, slurry concentration, maximum gangue particle size, and HPMC dosage need to be considered to optimize slurry performance in practical applications.

## 4. Engineering Application

### 4.1. Project Background

The annual discharge of gangue amounts to approximately 2 million tons in mine A located in Ordos City, Inner Mongolia Autonomous Region. The government-approved dumping gully for gangue is nearing full capacity. Consequently, the mine has planned to adopt adjacent backfilling technology for gangue disposal. Boreholes are drilled through coal pillars from the adjacent mining face’s haulage roadway to backfill the goaf.

**Figure 11:**
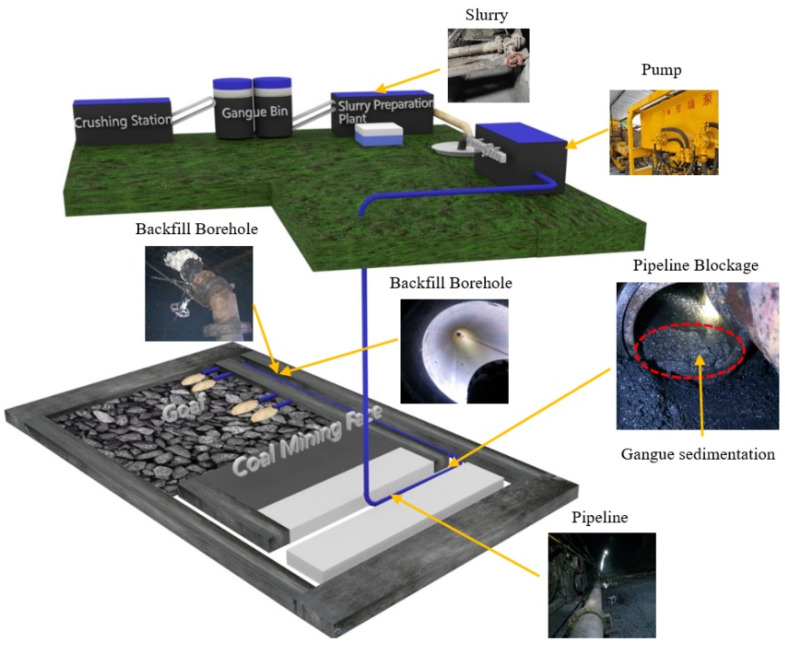
Layout of the backfilling system.

Figure 11 shows the system layout. This scheme utilizes gangue slurry with a maximum crushed particle size of 3 mm and 78% concentration. The control panel shows a sudden pipeline pressure surge 1 h after initiating the first backfilling operation, alongside a sharp increase in pump pressure. On-site inspection reveals a pipeline blockage in the section between the central haulage roadway and the intake airway, with the blocked section extending approximately 200 m. Upon dismantling the pipeline, the slurry in the blocked section has segregated, with a large amount of coarse particles settling at the bottom of the pipe.

### 4.2. Slurry Ratio Optimization

**Figure 12:**
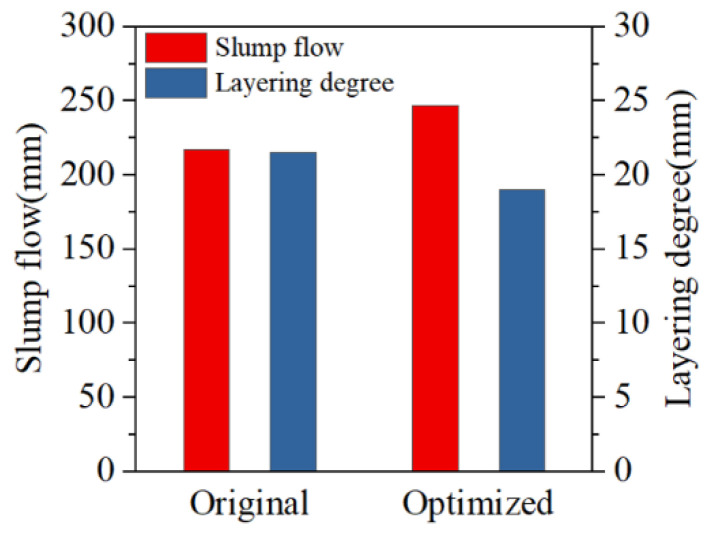
Performance comparison of the slurry before and after optimization.

Pipeline blockage is caused by slurry segregation, which necessitates an optimization of the slurry ratio. Based on the Talbol gradation formula (Equation (1)), the particle size distribution curve of the crushed gangue (with a maximum particle size of 3 mm) is fitted. n is 0.414, indicating that the gradation meets the requirements. Referring to the slurry ratio range established in Section 3.5, the optimized slurry ratio is determined as follows: a maximum gangue particle size of 3 mm, 0.045% HPMC dosage, and 77.5% slurry concentration. Performance tests of the optimized slurry (Figure 12) show an increase in slump flow by approximately 30 mm from 247 to 217 mm and a reduction in layering degree from 21.5 to 19.0 mm. Slurry stability improves significantly, with slurry segregation resolved.

### 4.3. Backfilling Effectiveness Inspection

**Figure 13:**
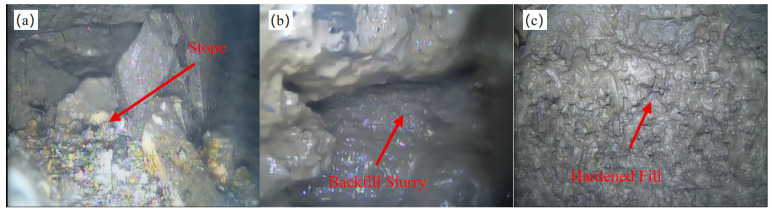
Borehole endoscopic observation: (**a**) Before backfilling, (**b**) During backfilling, (**c**) After backfilling.

The slurry exhibits excellent stability with no sedimentation of coarse gangue particles during the backfilling process. The pipeline remains unobstructed without any blockage. The slurry demonstrates outstanding fluidity in the goaf, with a maximum single-hole grouting volume of 2200 m^3^. Observations are performed using a borehole inspection camera (Jining Huakuang Machinery Equipment Co., Ltd., Jining City, China) at a location 60 m from the grouting hole (Figure 13). The borehole wall consists of fractured and loose rock fragments, and voids are filled with the backfill material, which demonstrates that the maximum diffusion radius of the slurry exceeds 60 m.

## 5. Conclusions

The work investigated the effects of X_1_, X_2_, and X_3_ on gangue slurry stability and fluidity using RSM. Response surface regression models were established to correlate stability/fluidity with these factors, which could obtain an optimal gangue slurry ratio. Water distribution and microstructural characteristics of the slurry were analyzed using LF-NMR, SEM, and PLM, elucidating the HPMC action mechanism. Industrial field tests validated HPMC’s effectiveness in regulating gangue slurry performance. The main conclusions are as follows.

(1) Slurry’s layering degree and slump flow decreased with increased X_1_ and X_3_; they decreased with reduced X_2_. Concentration exhibited the most significant influence, followed by maximum particle size and HPMC dosage. HPMC enhanced slurry stability while minimally compromising fluidity. X_1_X_2_ and X_1_X_3_ demonstrated significant effects on layering degree and slump flow. X_2_X_3_ significantly affected the layering degree but showed negligible influence on slump flow.
(2) The response surface regression models for slump flow and layering degree demonstrated excellent reliability (R^2^ > 0.99; *p* < 0.0001). The response surface regression model yielded an optimal gangue slurry ratio, with a gradation index of 0.40–0.428. Different gangue gradations corresponded to specific concentrations and HPMC dosages.
(3) Increased slurry concentration or reduced maximum gangue particle size significantly decreased interparticle spacing. Slurry stability was enhanced by intensifying the interparticle friction force. However, this process reduced free water content, which substantially impaired slurry fluidity. Upon HPMC addition, its molecular chains facilitated the formation of a 3D flocculation network through adsorption-bridging effects with fine particles. This mechanism inhibited coarse particle sedimentation and improved slurry stability. Meanwhile, HPMC addition converted only a minimal amount of free water to floc water, which exerted a limited negative impact on fluidity.
(4) Based on the response surface regression model, the gangue slurry ratio for mine A was optimized. The improved slurry demonstrated remarkable performance enhancement, with an increase of 30 mm in slump flow and a reduction of 2.5 mm in layering degree. The slurry filling process was free of pipe blockage, achieving a maximum spread radius of over 60 m and a maximum single-borehole filling volume of 2200 m^3^. This confirmed the effectiveness of HPMC in controlling gangue slurry performance.

## Figures and Tables

**Table 1:** Experimental design scheme.

Independent Variable	Horizontal Encoding		
	−1	0	1
X_1_	76	77	78
X_2_	2	3	4
X_3_	0.0	0.03	0.06

**Table 2:** Experimental results.

Serial No.	Concentration/%	Maximum Gangue Particle Size/mm	HPMC Dosage/%	Layering Degree/mm	Slump Flow/mm
1	78	4	0.03	241	21.5
2	77	2	0.06	206	14.2
3	76	2	0.03	264	25
4	76	3	0	297	33.3
5	76	4	0.03	298	33
6	77	3	0.03	268	23.5
7	78	2	0.03	153	12.1
8	77	4	0.06	275	23.5
9	77	2	0	244	22.9
10	76	3	0.06	269	25.5
11	77	3	0.03	272	23.5
12	77	3	0.03	271	23.7
13	78	3	0	216	21.4
14	78	3	0.06	182	12.4
15	77	4	0	301	31.3

**Table 3:** Variance analysis of the response surface regression model.

Source	Layering Degree		Slump Flow	
	*F*	*p*	*F*	*p*
Model	3537.4	<0.0001	1331.97	<0.0001
X_1_-X_1_	16,197.08	<0.0001	6318.81	<0.0001
X_2_-X_2_	8177.06	<0.0001	3442.39	<0.0001
X_3_-X_3_	7359.89	<0.0001	888.58	<0.0001
X_1_X_2_	26.02	0.0038	326.42	<0.0001
X_1_X_3_	19.12	0.0072	4.03	0.101
X_2_X_3_	10.75	0.022	16.12	0.0102
X_1_^2^	11.85	0.0184	906.56	<0.0001
X_2_^2^	34.72	0.002	103.62	0.0002
X_3_^2^	5.72	0.0622	57.88	0.0006

Note: *p* < 0.05 indicates a significant factor effect, while *p* > 0.05 suggests a non-significant effect.

**Table 4:** Slurry ratio validation.

Group No.	Slurry Concentration/%	Maximum Size/mm	HPMC Dosage/%	Measured Slump Flow/mm	Predicted Slump Flow/mm	Measured Layering Degree/mm	Predicted Layering Degree/mm
S_1_	77.25	2.5	0.045	217.7	228.9	16.8	17.4
S_2_	77.50	3.0	0.045	238.3	233.8	19.1	18.2
S_3_	77.25	3.0	0.050	237.8	245.0	18.3	19.1
S_4_	77.80	3.5	0.045	241.4	231.5	18.9	18.5

## Data Availability

The original contributions presented in this study are included in the article. Further inquiries can be directed to the corresponding author.

## Abbreviations

CG, Coal gangue; HPMC, Hydroxypropyl methyl cellulose; MS, molecular structural; PSD, particle size distribution; XRD, X-ray Diffraction; BBD, Box-Behnken Design; LF-NMR, Low-Field Nuclear Magnetic Resonance; SEM, Scanning Electron Microscope; PLM, Polarized Light Microscopy
